# Association Between Different Thyroid-Stimulating Hormone Levels and Macrovascular Complications in Subclinical Hypothyroidism Patients With Type 2 Diabetes Mellitus

**DOI:** 10.7759/cureus.79186

**Published:** 2025-02-17

**Authors:** N M Motachim Mahmud, Dhierin R Jagdewsing, Xiaochen Ji, Ibrahim Harine, Bahassane Adjibou, Noor Safra C Fahmy, Thomas Juby, Rafiul I Shuvo, Ausraful Alam, Saudeya Sarmin

**Affiliations:** 1 Department of Endocrinology and Metabolism, The Second Affiliated Hospital of Dalian Medical University, Dalian, CHN; 2 Department of General Surgery, Dalian Medical University, Dalian, CHN; 3 Department of Emergency Medicine, Chad-China Friendship Hospital, N’Djamena, TCD; 4 Department of Internal Medicine, Hospital el Maarouf, Moroni, COM; 5 Department of Internal Medicine, Dalian Medical University, Dalian, CHN; 6 Department of General Surgery, The First Affiliated Hospital of Dalian Medical University, Dalian, CHN; 7 Department of Internal Medicine, Bangabandhu Sheikh Mujib Medical University (BSMMU), Dhaka, BGD; 8 Department of Community Opthalmology, Bangabandhu Sheikh Mujib Medical University (BSMMU), Dhaka, BGD; 9 Department of Radiology and Imaging, Pabna Medical Consultation Center, Pabna, BGD

**Keywords:** cerebrovascular disease (cvd), coronary artery disease (cad), lipid metabolism, macrovascular complications, peripheral artery disease (pad), subclinical hypothyroidism, thyroid-stimulating hormone (tsh), type 2 diabetes mellitus

## Abstract

Aim: Subclinical hypothyroidism (SCH) is frequently observed in patients with type 2 diabetes mellitus (T2DM) and may exacerbate macrovascular complications due to its impact on metabolic and thyroid function parameters. This study aims to explore the association between varying levels of thyroid-stimulating hormone (TSH) and the risk of macrovascular complications, alongside analyzing key metabolic, demographic, and clinical factors in T2DM patients with SCH.

Methods: A retrospective study was conducted at the Second Affiliated Hospital of Dalian Medical University, and data was collected from 2017 to 2023. According to their TSH levels, 305 patients were divided into three groups, which were T2DM mild SCH (TSH 4.34-6.9 mIU/L), T2DM moderate SCH (TSH 7.0-9.9 mIU/L), and T2DM severe SCH (TSH levels of 10.0 mIU/L or higher). The chi-square test was used for categorical variables, while one-way analysis of variance (ANOVA) was used for continuous variables. Univariate and multivariate binary logistic regression analysis was performed to determine the risk of macrovascular complications. Further, a statistical significance was set at p <0.05.

Results: Patients with severe SCH had the highest incidence of macrovascular complications, 19 (90.5%), followed by moderate SCH 38 (80.9%) and mild SCH 142 (59.9%) (p < 0.001). Multivariate analysis revealed a 4.35-fold increased risk (OR: 4.352, 95% CI: 1.761-10.754, p = 0.001) for macrovascular complications in moderate SCH and a 6.08-fold increased risk (OR: 6.075, 95% CI: 1.202-30.715, p = 0.029) in severe SCH compared to mild SCH. Age group 65 and older, male sex, and severe SCH were significant predictors of macrovascular complications. Peripheral artery disease (PAD) and coronary artery disease (CAD) were particularly associated with severe SCH (OR: 5.913, p < 0.001; OR: 3.268, p = 0.013, respectively).

Conclusion: T2DM patients with severe or moderate SCH are at significantly higher risk of macrovascular complications, especially PAD and CAD. Timely intervention and close monitoring of TSH levels, particularly in older and male patients, are essential to mitigate these risks.

## Introduction

Diabetes mellitus is one of the most common endocrine diseases in the world, with type 2 diabetes mellitus (T2DM) rates increasing and the number of affected people expected to reach 640 million by 2040 [1]. This disorder may be accompanied by other thyroid disorders, such as subclinical hypothyroidism (SCH), which worsens the prognosis of T2DM patients. It is thus important to establish the correlation between thyroid function, particularly thyroid-stimulating hormone (TSH), and macrovascular complications in patients with T2DM. Cardiovascular diseases, cerebrovascular diseases (CVDs), and peripheral vascular diseases (PVDs) are some of the macrovascular complications that continue to be associated with high mortality and morbidity in T2DM patients [2]. A study on diabetic patients showed a two- to four-fold increased probability of cardiovascular diseases and an independent risk factor for CVDs and myocardial infarction (MI) [3]. A large proportion of thyroid disorders in T2DM patients is related to SCH [4,5]. SCH has been reported to be present in 4-17% of the population with diabetes [6]. In typical T2DM patients often TSH levels increase, which may have a strong impact on the cardio-metabolic risk factors, suggesting that SCH is associated with adverse cardiovascular prognosis [7]. The combined impact of T2DM and SCH on lipid profile increases the risk of CVD; T2DM affects lipid metabolism and results in increased levels of low-density lipoprotein cholesterol (LDL-C) and triglycerides (TG) [8]. SCH, with increased TSH levels, continues the further progression of lipid metabolism disturbances, increasing the risk of atherosclerotic processes and cardiovascular events [9].

It is essential to obtain a clear and detailed picture of TSH and the differences and interconnections between T2DM patients with SCH and other comorbidities, which will fill the gaps in the existing knowledge of SCH-T2DM comorbidity and provide crucial information regarding risk factors, clinical significance, and interventions. SCH is a thyroid dysfunction characterized by increased TSH levels with normal free triiodothyronine (FT3) and free thyroxine (FT4) levels, affecting 4-20% of the population [10]. T2DM, which is the most common metabolic disorder, is becoming more prevalent, especially among individuals with inactive lifestyles and poor eating habits [11]. SCH has been known to relate to T2DM, and SCH can be used to predict the occurrence of T2DM [12]. Macrovascular complications of T2DM are CVD, stroke, and PVD, which are associated with atherosclerosis [13]. Peripheral artery disease (PAD) impacts the arteries that supply blood to the extremities, and T2DM patients are at increased risk due to atherosclerosis, endothelial dysfunction, and peripheral neuropathy [14]. Another study provided evidence that SCH in patients with T2DM is associated with a high prevalence of coronary heart disease [15]. On the other hand, previous studies have observed inconsistent results regarding the relationship between TSH levels and cardiovascular events in T2DM patients, suggesting that more research is required to fill knowledge gaps and provide definitive recommendations for managing TSH levels in T2DM patients [16,17]. Therefore, it is important to do this research and better understand the link between raised TSH and macrovascular complications in T2DM patients so that better clinical management strategies can be implemented to enhance the health of these patients.

## Materials and methods

This study is based on the patient data from the Second Affiliated Hospital of Dalian Medical University, China, which included T2DM and SCH patients during the years 2017-2023. There were 305 participants included in this study, all of whom were T2DM patients diagnosed with SCH, subdivided by TSH values. Since TSH levels of T2DM with mild SCH patients in the Second Affiliated Hospital of Dalian Medical University started from 4.34 mIU/L, the range of mild TSH was then set to 4.34-6.9 mIU/L. Further, in patients with T2DM and moderate SCH, the TSH levels were set to 7.0-9.9 mIU/L. At last, for the patients with T2DM and severe SCH, the TSH levels were set to 10 mIU/L and higher. This study design allowed for an in-depth investigation of the long-term association between T2DM, SCH, and macrovascular complications.

Patient selection

The inclusion criteria included participants aged between 25 and 90 with confirmed T2DM and laboratory confirmation of thyroid hormone abnormalities, as well as patients with macrovascular complications, including CVD, coronary artery disease (CAD), and PAD. This study excluded patients with type 1 diabetes mellitus, other thyroid diseases (Graves’ disease or thyroid carcinoma), autoimmunity disorders, malignant tumors, infectious diseases, pregnancy, and mental illnesses. Further, patients with alcohol intake, a smoking history in the last 10 years, or a body mass index (BMI) above 40 were also excluded from the study as these factors independently cause macrovascular disease (MVC).

Data collection

TSH levels were the main independent variables divided into mild, moderate, and severe SCH. The main dependent variable was the presence of macrovascular complications. Macrovascular complications in this study included PAD, CVD, and CAD. Conditions such as stroke and transient ischemic attack (TIA), carotid artery disease, cerebral aneurysm including CVD, and symptoms like angina (stable and unstable), MI, heart failure, and sudden cardiac arrest come under CAD, symptoms such as intermittent claudication, critical limb ischemia, acute limb ischemia, and Buerger’s disease are included in the study as PAD. Other parameters included were age, gender, BMI, duration of T2DM, systolic and diastolic blood pressure, lipid profile, thyroid profile (FT3 and FT4), fasting blood glucose, hemoglobin A1C (HbA1C), and hospitalization days.

Data analysis

The data was analyzed using IBM SPSS Statistics for Windows, Version 29.0.2 (Released 2023; IBM Corp., Armonk, NY). Chi-square analyses were used to test the significance of the association between categorical variables, while one-way analysis of variance (ANOVA) was used to compare the mean values of continuous variables. Univariate and multivariate binary logistic regression analysis was used for the risk of macrovascular complications and their subtypes comprising PAD, CAD, and CVD. Further, a statistical significance was set at p <0.05.

## Results

Table 1 compares various clinical and biochemical parameters among patients with T2DM categorized by the severity of SCH into mild (n = 237), moderate (n = 47), and severe (n = 21) groups. Notably, the prevalence of macrovascular complications increases with SCH severity, reported at 142 (59.9%) in the mild group, 38 (80.9%) in the moderate group, and 19 (90.5%) in the severe group (p < 0.001). Additionally, significant differences are observed in thyroid hormone levels: mean FT3 levels decrease from 4.58 ± 0.57 pmol/L in mild SCH to 4.00 ± 0.49 pmol/L in severe SCH (p < 0.001) and mean FT4 levels decline from 14.86 ± 2.12 pmol/L to 13.23 ± 1.52 pmol/L across the same groups (p = 0.002). Lipid profiles also show significant variations, with high-density lipoprotein cholesterol (HDL-C) levels decreasing from 1.20 ± 0.31 mmol/L in mild SCH to 1.02 ± 0.16 mmol/L in severe SCH (p = 0.025) and total cholesterol (TC) levels increasing from 5.10 ± 1.21 mmol/L to 5.83 ± 2.34 mmol/L (p = 0.023). Other parameters, including age, gender distribution, BMI, duration of T2DM, blood pressure, LDL-C, TG, fasting blood glucose (FBG), HbA1C, and length of hospital stay, do not exhibit statistically significant differences across SCH severity levels.

**Table 1:** Comparison of general data among various levels of SCH severity individuals with T2DM (N = 305) T2DM: type 2 diabetes mellitus; SCH: subclinical hypothyroidism; BMI: body mass index; BP: blood pressure; FT3: free triiodothyronine; FT4: free thyroxine; LDL-C: low-density lipoprotein cholesterol; HDL-C: high-density lipoprotein cholesterol; TC: total cholesterol; TG: triglycerides; FBG: fasting blood glucose; HbA1C: hemoglobin A1C.

Variable	T2DM Mild SCH (n = 237)	T2DM Moderate SCH (n = 47)	T2DM Severe SCH (n = 21)	p-Value
Macrovascular complications, n (%)	142 (59.9)	38 (80.9)	19 (90.5)	<0.001
Age (years), mean ± SD	62.80 ± 12.61	62.02 ± 13.1	68.24 ± 11.70	0.140
Gender, n (%)				0.685
Female	151 (63.7)	33 (70.2)	14 (66.7)	
Male	86 (36.3)	14 (29.8)	7 (33.3)	
BMI, mean ± SD	26.31 ± 3.87	25.49 ± 3.76	25.82 ± 3.36	0.378
T2DM duration (years), mean ± SD	10.00 ± 7.61	8.78 ± 8.24	9.48 ± 7.00	0.606
BP systolic, mean ± SD	141.81 ± 18.94	142.40 ± 20.15	143.38 ± 19.79	0.824
BP diastolic, mean ± SD	79.60 ± 11.09	81.04 ± 12.46	77.81 ± 10.91	0.808
FT3 (pmol/L), mean ± SD	4.58 ± .57	4.61 ± .060	4.00 ± .49	<0.001
FT4 (pmol/L), mean ± SD	14.86 ± 2.12	14.44 ± 1.78	13.23 ± 1.52	0.002
LDL-C (mmol/L), mean ± SD	2.82 ± 0.91	3.13 ± 1.10	3.13 ± 1.79	0.089
HDL-C (mmol/L), mean ± SD	1.20 ± 0.31	1.15 ± 0.26	1.02 ± 0.16	0.025
TC (mmol/L), mean ± SD	5.10 ± 1.21	5.49 ± 1.60	5.83 ± 2.34	0.023
TG (mmol/L), mean ± SD	2.14 ± 1.99	2.47 ± 3.13	1.88 ± 0.97	0.518
FBG (mmol/L), mean ± SD	8.80 ± 3.44	8.24 ± 3.26	9.58 ± 3.79	0.317
HbA1C (%), mean ± SD	10.25 ± 12.28	8.60 ± 2.28	8.97 ± 2.27	0.586
Hospital stay (days), mean ± SD	7.19 ± 1.47	7.55 ± 1.73	7.43 ± 1.54	0.283

In the aspect of macrovascular complications, we observe compelling occurrence rates of PAD, CAD, and CVD across different SCH severity categories. The number of patients with mild SCH was 58 (24.5%), with moderate SCH being 15 (31.9%), and with severe SCH, 14 (66.7%) exhibited PAD. The trend relates to PAD occurrence and SCH severity classes, with a significant p-value of <0.001. The incidence of CAD also widens from 61 (25.7%) in people who suffer from mild SCH to 12 (57.1%) in those with severe SCH, and the people with moderate SCH stand for 13 (27.7%). Nevertheless, the p-value of 0.009 is much like the PAD for the three categories of SCH. It is also statistically significant. In patients with mild SCH, the prevalence of CVD was 66 (27.8%), and it grew to 18 (38.3%) in the moderate SCH and advanced to 11 (52.4%) in the severe SCH. A p-value of 0.034 represents the significance of the difference between the occurrence of cerebral disease and the different SCH severity categories (Table 2).

**Table 2:** Prevalence of PAD, CAD, and CVD among T2DM patients with different levels of SCH (N = 305) T2DM: type 2 diabetes mellitus; SCH: subclinical hypothyroidism; PAD: peripheral artery disease; CAD: coronary artery disease; CVD: cerebrovascular disease.

Variable	T2DM Mild SCH (n = 237)	T2DM Moderate SCH (n = 47)	T2DM Severe SCH (n = 21)	p-Value
PAD, n (%)	58 (24.5)	15 (31.9)	14 (66.7)	<0.001
CAD, n (%)	61 (25.7)	13 (27.7)	12 (57.1)	0.009
CVD, n (%)	66 (27.8)	18 (38.3)	11 (52.4)	0.034

Table 3 shows the univariate and multivariate logistic regression results with significant predictors. In the univariate analysis, patients with T2DM + severe SCH show significant findings compared to those with mild SCH (OR: 6.356, 95% CI: 1.447-27.920, p= 0.014), and moderate SCH category also shows significant findings (OR: 2.825, 95% CI: 1.306-6.111, p =0.008). Age group 65 and older had the highest odds compared to <45 years (OR = 138, 95% CI: 4.032-30.766, p < 0.001), and the 45-64-year age group also showed elevated odds (OR = 3.731, 95% CI: 1.387-10.034, p = 0.009). On the other hand, male sex shows significant results (OR: 3.630, 95% CI: 2.053-6.417, p < 0.001). Multivariate analysis strongly supports these findings: T2DM with severe SCH shows higher odds (OR: 6.075, 95% CI: 1.202-30.715, p = 0.029), as well as moderate SCH (OR: 4.352, 95% CI: 1.761-10.754, p = 0.001), age 65 and older (OR: 13.492, 95% CI: 4.326-42.077, p < 0.001), and age 45-64 (OR: 4.531, 95% CI: 1.516-13.542, p = 0.007). Male sex also shows more significant findings (OR: 5.488, 95% CI: 2.686-11.212, p < 0.001). It is clear from these results that different TSH levels, age, and sex are the most significant predictors.

**Table 3:** Univariate and multivariate binary logistic regression analysis for the risk of macrovascular complications (N = 305) T2DM: type 2 diabetes mellitus; SCH: subclinical hypothyroidism; OR: odds ratio; CI: confidence interval; BMI: body mass index; FT3: free triiodothyronine; FT4: free thyroxine; LDL-C: low-density lipoprotein cholesterol; HDL-C: high-density lipoprotein cholesterol; TC: total cholesterol; TG: triglycerides.

Univariate			
Variable	OR	95% CI	p-Value
T2DM + moderate SCH vs mild SCH	2.825	1.306-6.111	0.008
T2DM + severe SCH vs mild SCH	6.356	1.447-27.920	0.014
Age group: 45-64 years vs <45 years	3.731	1.387-10.034	0.009
Age group: 65 years and older vs <45 years	11.138	4.032-30.766	<0.001
Male vs Female	3.630	2.053-6.417	<0.001
BMI ≥25 vs <25	0.847	0.522-1.373	0.501
FT3 (pmol/L)	0.754	0.501-1.136	0.177
FT4 (pmol/L)	0.961	0.858-1.077	0.497
LDL-C (mmol/L)	0.854	0.680-1.074	0.177
HDL-C (mmol/L)	0.658	0.301-1.438	0.294
TC (mmol/L)	0.910	0.769-1.077	0.271
TG (mmol/L)	0.854	0.680-1.074	0.177
Multivariate			
Variable	OR	95% CI	p-Value
T2DM + moderate SCH vs mild SCH	4.352	1.761-10.754	0.001
T2DM + severe SCH vs mild SCH	6.075	1.202-30.715	0.029
Age group: 45-64 years vs <45 years	4.531	1.516-13.542	0.007
Age group: 65 years and older vs <45 years	13.492	4.326-42.077	<0.001
Sex: Male	5.488	2.686-11.212	<0.001

The results of the multivariate binary logistic regression analysis for subtypes showed that sex, age, and SCH severity were independent predictors of macrovascular complications in patients with T2DM (Table 4). Male sex consistently emerged as a significant risk factor, with higher odds of developing PAD (OR: 1.913, p = 0.018), CVD (OR: 2.252, p = 0.003), and CAD (OR: 1.753, p = 0.041). Severe SCH was significantly associated with PAD (OR: 5.913, p < 0.001) and CAD (OR: 3.268, p= 0.013) but had only a borderline significance about CVD (OR: 2.463, p = 0.061); moderate SCH did not reveal significant effect. Age also played an important role, as the 65 and older age group exhibited markedly increased risks for CVD (OR: 9.144, p = 0.004) and CAD (OR: 1.902, p = 0.017) compared to younger groups, though it had no significant effect on PAD. These observations point to the fact that macrovascular complications are more common in male patients, elderly patients, and those with severe SCH in T2DM.

**Table 4:** Multivariate binary logistic regression analysis for macrovascular complications subtype (N = 305) T2DM: type 2 diabetes mellitus; SCH: subclinical hypothyroidism; PAD: peripheral artery disease; CVD: cerebrovascular disease; CAD: coronary artery disease; OR: odds ratio; CI: confidence interval.

Variable	OR	95% CI	p-Value
PAD			
T2DM + moderate SCH vs mild SCH	1.542	0.766-3.106	0.225
T2DM + severe SCH vs mild SCH	5.913	2.202-15.877	<0.001
Age group: 45-64 years vs <45 years	1.141	0.388-3.353	0.811
Age group: 65 years and older vs <45 years	2.308	0.805-6.617	0.120
Sex: Male vs Female	1.913	1.116-3.280	0.018
CVD			
T2DM + moderate SCH vs mild SCH	1.751	0.881-3.480	0.110
T2DM + severe SCH vs mild SCH	2.463	0.960-6.319	0.061
Age group: 45-64 years vs <45 years	4.546	1.003-20.601	0.050
Age group: 65 years and older vs <45 years	9.144	2.041-40.966	0.004
Sex: Male vs Female	2.252	1.330-3.813	0.003
CAD			
T2DM + moderate SCH vs mild SCH	1.155	0.557-2.394	0.699
T2DM + severe SCH vs mild SCH	3.268	1.279-8.351	0.013
Age group: 65 years and older vs 45-64 years	1.902	1.120-3.232	0.017
Sex: Male vs Female	1.753	1.022-3.008	0.041

Table 5 shows that CAD, PAD, and CVD are distributed substantially differently among age groups, sexes, and TSH levels and are associated with these risk factors. Age shows significant correlation with macrovascular complication subtypes, p < 0.001 for CAD and CVD and p = 0.008 for PAD, respectively. The age group 65 years and older shows the highest prevalence, 55 (38.5%) for CAD, 53 (37.1%) for PAD, and 59 (41.3%) for CVD; the lowest prevalence was found in the <45 years age group. Sex differences also show notable findings, especially for PAD (p = 0.024) and CVD (p = 0.006), where male patients demonstrated higher prevalences, 39 (36.4%) and 44 (41.1%), respectively, compared to female patients, 48 (24.2%) and 51 (25.8%), respectively. Although the incidence of CAD was more common among male patients, 37 (34.6%), than female patients, 49 (24.7%), the difference was not significant (p = 0.069). All three complications were significantly associated with TSH levels, with the most prevalent in the severe SCH group with CAD (12 (57.1%), p = 0.009), PAD (14 (66.7%), p < 0.001), and CVD (11 (52.4%), p = 0.034), and the least in the mild SCH patients. Therefore, these findings suggest that advanced age, male sex, and higher TSH are independent risk factors for macrovascular complications in T2DM patients requiring a targeted intervention in high-risk populations.

**Table 5:** Association of age group, sex, and TSH levels with prevalence of CAD, PAD, and CVD (N = 305) CAD: coronary artery disease; PAD: peripheral artery disease; CVD: cerebrovascular disease; TSH: thyroid-stimulating hormone.

Variable	CAD	p-Value	PAD	p-Value	CVD	p-Value
Age group		<0.001		0.008		<0.001
<45 years	0 (0.0)		5 (19.2)		2 (7.7)	
45-64 years	31 (22.8)		29 (21.3)		34 (25.0)	
65 years and older	55 (38.5)		53 (37.1)		59 (41.3)	
Sex		0.069		0.024		0.006
Male	37 (34.6)		39 (36.4)		44 (41.1)	
Female	49 (24.7)		48 (24.2)		51 (25.8)	
TSH level		0.009		<0.001		0.034
Mild	61 (25.7)		58 (24.5)		66 (27.8)	
Moderate	13 (27.7)		15 (31.9)		18 (38.3)	
Severe	12 (57.1)		14 (66.7)		11 (52.4)	

**Figure 1:**
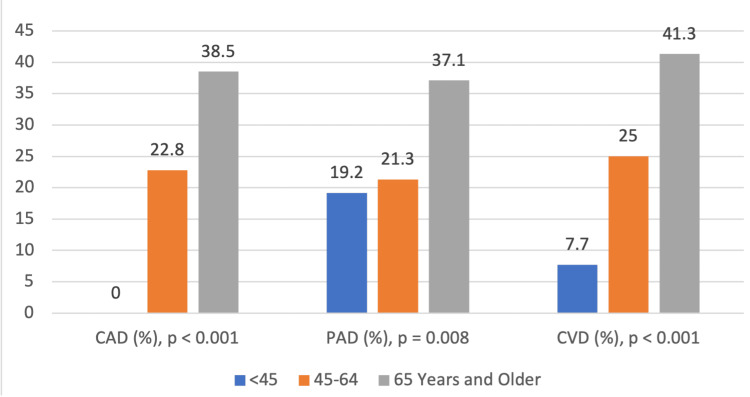
Prevalence of CAD, PAD, and CVD across different age groups in T2DM patients with SCH CAD: coronary artery disease; PAD: peripheral artery disease; CVD: cerebrovascular disease.

Figure 1 presents the prevalence of PAD, CAD, and CVD among T2DM patients with varying degrees of SCH (mild, moderate, and severe). The prevalence of PAD significantly increases with SCH severity, rising from 24.5% in mild SCH to 31.9% in moderate SCH and 66.7% in severe SCH (p < 0.001). A similar trend is observed for CAD (25.7%, 27.7%, and 57.1%, respectively; p = 0.009) and CVD (27.8%, 38.3%, and 52.4%, respectively; p = 0.034). The statistically significant p-values indicate a strong association between increasing SCH severity and higher prevalence of PAD, CAD, and CVD in T2DM patients.

**Figure 2:**
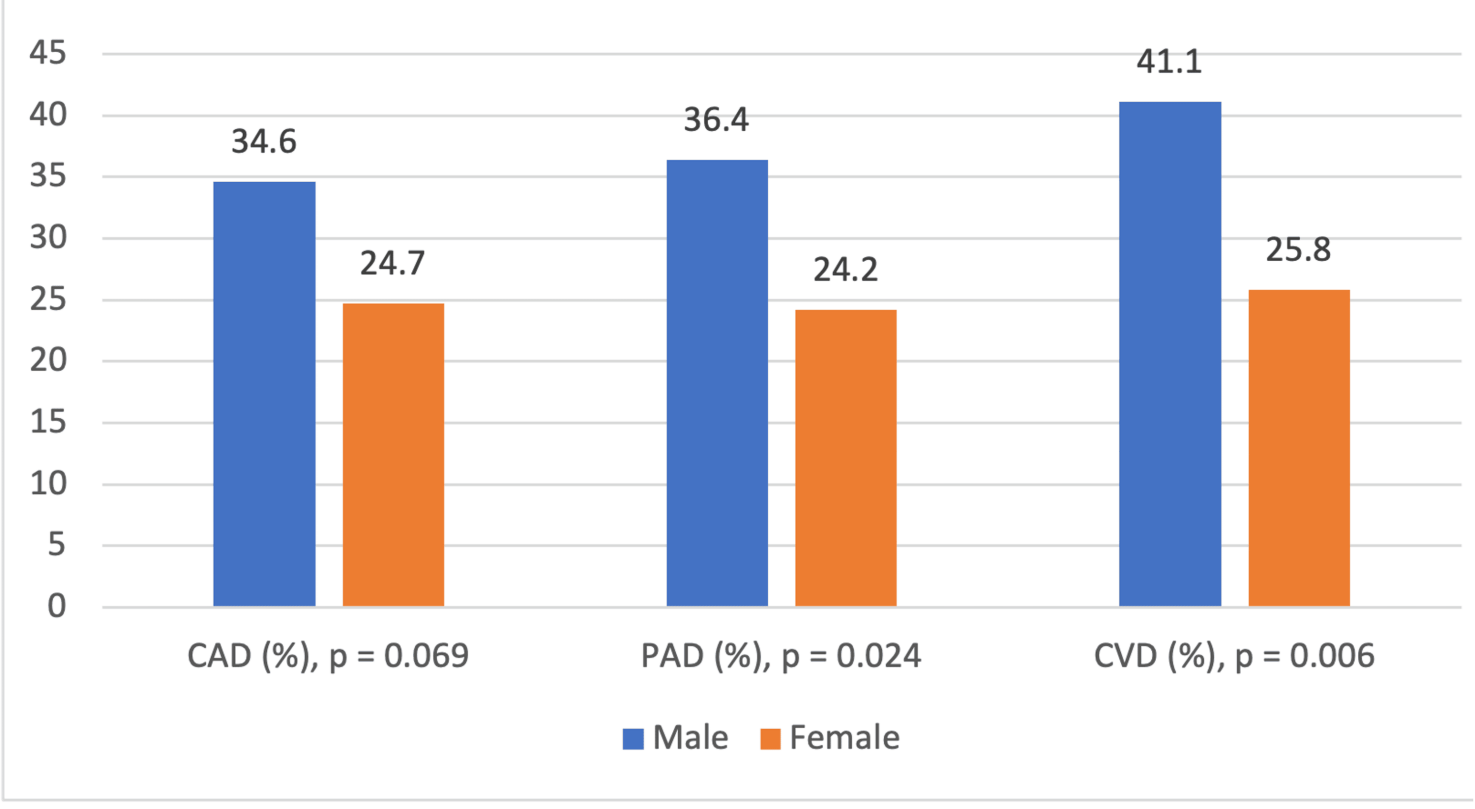
Prevalence of CAD, PAD, and CVD among male and female T2DM patients with SCH CAD: coronary artery disease; PAD: peripheral artery disease; CVD: cerebrovascular disease.

Figure 2 compares the prevalence of CAD, PAD, and CVD between male and female patients. For CAD, male patients show a higher prevalence of 34.6% compared to 24.7% in female patients, though this difference is not statistically significant (p = 0.069). However, significant differences are observed for PAD and CVD, with male patients having a prevalence of 36.4% and 41.1%, respectively, compared to 24.2% and 25.8% in female patients (p = 0.024 for PAD and p = 0.006 for CVD). These findings highlight that male patients have a consistently higher prevalence of PAD and CVD compared to female patients, with statistically significant differences.

**Figure 3:**
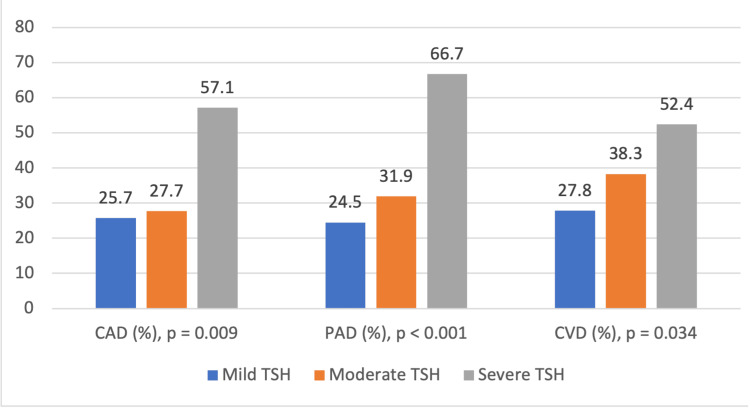
Prevalence of CAD, PAD, and CVD by different TSH levels in T2DM patients with SCH CAD: coronary artery disease; PAD: peripheral artery disease; CVD: cerebrovascular disease; TSH: thyroid-stimulating hormone; T2DM: Type 2 diabetes mellitus.

Figure 3 shows the prevalence of CAD, PAD, and CVD across patients with varying levels of TSH categorized as mild, moderate, and severe. The prevalence of CAD increases from 25.7% in mild TSH to 27.7% in moderate TSH and 57.1% in severe TSH (p = 0.009). Similarly, the prevalence of PAD rises significantly from 24.5% in mild TSH to 31.9% in moderate TSH and 66.7% in severe TSH (p < 0.001). For CVD, the prevalence also increases from 27.8% in mild TSH to 38.3% in moderate TSH and 52.4% in severe TSH (p = 0.034). These findings highlight a strong association between increasing TSH levels and the prevalence of CAD, PAD, and CVD.

## Discussion

T2DM and the various stages of SCH were found to have a very close relationship regarding the macrovascular complications of the patients. In our study, we found that the severe SCH cohort had the highest macrovascular event of 19 (90.5%), approximately 6.4 times higher odds of developing macrovascular complications compared to those with mild SCH. The results obtained from the univariate and multivariate logistic regression analysis support the importance of TSH levels, age, and sex in determining the outcomes investigated. Elevated TSH levels, particularly in the severe SCH category, were strongly associated with increased odds of the outcome in both univariate (OR: 6.356, 95% CI: 1.447-27.920, p = 0.014) and multivariate analyses (OR: 6.075, 95% CI: 1.202-30.715, p = 0.029). A study that outlined macrovascular complications of diabetes demonstrated that the prevalence of PAD was significantly higher in patients with SCH compared to euthyroid T2DM patients [12]. Others found that SCH has been associated with increased cardiovascular mortality due to adverse effects mainly on lipids and blood pressure and also evidence that SCH, especially in patients with TSH >10 mU/L, may increase cardiovascular risk [18].

Regression analysis was not statistically significant for FT3, FT4, HDL-C, LDL-C, TC, and TG in our study, which could be due to the small sample size of these variables or their lesser correlation with the outcomes under the study. Although these results do not negate the clinical relevance of these factors, they indicate that their expression may be more conditional or influenced by other factors. Further, another important predictor was age; the patients aged 65 years and older had significantly higher risks of developing CVD or CAD than those of a younger age. These findings indicate that age is an important modifier most likely because of cumulative vascular injury and other diseases in the elderly population. Age was not associated with the development of PAD, which may indicate that the processes that lead to this complication are different. Taken together, these data highlight the need to take into account sex, age, and SCH severity when evaluating and treating T2DM patients at risk of macrovascular complications.

The findings of the multivariate binary logistic regression analysis of subgroups reveal significant risk factors that led to macrovascular complications in T2DM patients. SCH was found to be independently associated with PAD and CAD. The result of the study is also in line with the research work, which states that the pathological condition of PAD has significantly increased in patients with SCH against the euthyroid individuals [19]. Such discovery is in line with our study, which showed that individuals with SCH had high chances of CAD, particularly those with higher TSH levels. Furthermore, in the SCH-CAD risk study conducted, there was an association between heart disease and SCH. This study also follows the same trend that there is a stronger tendency to develop strokes and heart diseases in patients with SCH, mainly as exhaustion of TSH rises [20]. The majority of patients diagnosed with T2DM and SCH experienced MVC, specifically TIA/stroke (23.3%), angina pectoris (16.7%), PAD (10.0%), and abnormal lipid profile [17]. These findings provide significant evidence for our study. Another research also noticed that SCH is a risk factor for ischemic stroke and affects recovery [21].

This study also found age as a significant factor; groups 45-64 years and 65 and older had significantly higher odds ratios than those below 45 years. The strongest association was observed in the 65 years and older age group (multivariate OR: 13.492, 95% CI: 4.326-42.077, p < 0.001), indicating the need for appropriate intervention in the elderly. This is in line with the previous research showing that older adults are at a higher risk of experiencing adverse health outcomes because of comorbidities, decreased physiological reserve capacity, and slow healing rates. A meta-analysis published in 2020 concluded that SCH in older age is a high-risk risk factor for the onset of macrovascular complications and mortality [22].

Sex was another significant predictor, with male patients demonstrating higher odds compared to female patients (multivariate OR: 5.488, 95% CI: 2.686-11.212, p < 0.001). This is in line with a study that suggests that male patients are at a higher risk of macrovascular complications with T2DM [23]. In general, the results of this study contribute to the understanding that factors like age, male sex, and severe TSH levels can increase the risk of macrovascular complications in T2DM patients with SCH.

Limitations and recommendations

This study provides valuable insights into the association between TSH levels and macrovascular complications in T2DM patients with SCH. However, certain limitations must be acknowledged. First, the retrospective single-center design may restrict the generalizability of the findings, as variations in healthcare settings, genetic predispositions, and environmental factors were not accounted for. Additionally, the sample size, particularly the severe SCH group (n = 21), was relatively small, which may affect the statistical power of the analysis. Another limitation is the cross-sectional nature of the study, which prevents conclusions about causality or the progression of macrovascular complications over time. Furthermore, some important confounders, such as inflammatory markers, thyroid hormone replacement therapy (THRT), and lifestyle factors, were not included in the analysis, which may influence the observed associations.

To address these limitations, future research should include prospective cohort studies to track TSH fluctuations over time and assess their long-term impact on macrovascular complications. Additionally, multicenter studies with larger, diverse populations can improve the validity of these findings. Investigating the potential benefits of THRT on cardiovascular outcomes in T2DM patients with SCH through randomized controlled trials is also recommended. Further studies should explore inflammatory and metabolic pathways, including oxidative stress markers, to better understand the mechanisms linking SCH, T2DM, and MVC. By addressing these gaps, future research can provide more robust clinical guidelines for the management of SCH in T2DM patients at risk for cardiovascular complications.

## Conclusions

Patients with T2DM and moderate or severe SCH are at a significantly higher risk of macrovascular complications, particularly PAD and CAD, compared to those with mild SCH. Elevated TSH levels strongly contribute to these risks, with older age and male sex further increasing the likelihood of adverse outcomes. These findings emphasize the importance of close monitoring and timely intervention for T2DM patients with elevated TSH levels. Tailored risk management strategies focusing on these high-risk groups can play a vital role in improving long-term vascular health and overall outcomes.
